# Discovery and validation of methylation signatures in circulating cell-free DNA for early detection of esophageal cancer: a case-control study

**DOI:** 10.1186/s12916-021-02109-y

**Published:** 2021-10-13

**Authors:** Guibin Qiao, Weitao Zhuang, Bo Dong, Chengcheng Li, Jiayue Xu, Guoqiang Wang, Liang Xie, Zihao Zhou, Dan Tian, Gang Chen, Jiming Tang, Haiyu Zhou, Dongkun Zhang, Ruiqing Shi, Rixin Chen, Weiqi Nian, Yuzi Zhang, Jing Zhao, Xiaofang Wen, Yu Xu, Bingsi Li, Zhihong Zhang, Shangli Cai, Xiaosong Ben, Yu Qi

**Affiliations:** 1 Department of Thoracic Surgery, Guangdong Provincial People’s Hospital, Guangdong Academy of Medical Sciences, 106 Zhongshan Second Road, Guangzhou, 510080 China; 2 Shantou University Medical College, Shantou, 515041 China; 3 Department of Thoracic Surgery, First Affiliated Hospital of Zhengzhou University, No.1 Jianshe East Road, Zhengzhou, 450052 Henan Province China; 4 Burning Rock Biotech, Guangzhou, Guangdong 510300 China; 5 Research Center of Medical Sciences, Guangdong Provincial People’s Hospital, Guangdong Academy of Medical Sciences, Guangzhou, 510080 China; 6 Phase I ward, Chongqing University Cancer Hospital, Chongqing, 400030 China

**Keywords:** Esophageal cancer, Methylation, Circulating cell-free DNA, Early detection, Liquid biopsy

## Abstract

**Background:**

Plasma cell-free DNA (cfDNA) methylation has shown promising results in the early detection of multiple cancers recently. Here, we conducted a study to investigate the performance of cfDNA methylation in the early detection of esophageal cancer (ESCA).

**Methods:**

Specific methylation markers for ESCA were identified and optimized based on esophageal tumor and paired adjacent tissues (*n* = 24). Age-matched participants with ESCA (*n* = 85), benign esophageal diseases (*n* = 10), and healthy controls (*n* = 125) were randomized into the training and test sets to develop a classifier to differentiate ESCA from healthy controls and benign esophageal disease. The classifier was further validated in an independent plasma cohort of ESCA patients (*n* = 83) and healthy controls (*n* = 98).

**Results:**

In total, 921 differentially methylated regions (DMRs) between tumor and adjacent tissues were identified. The early detection classifier based on those DMRs was first developed and tested in plasma samples, discriminating ESCA patients from benign and healthy controls with a sensitivity of 76.2% (60.5–87.9%) and a specificity of 94.1% (85.7–98.4%) in the test set. The performance of the classifier was consistent irrespective of sex, age, and pathological diagnosis (*P* > 0.05). In the independent plasma validation cohort, similar performance was observed with a sensitivity of 74.7% (64.0–83.6%) and a specificity of 95.9% (89.9–98.9%). Sensitivity for stage 0–II was 58.8% (44.2–72.4%).

**Conclusion:**

We demonstrated that the cfDNA methylation patterns could distinguish ESCAs from healthy individuals and benign esophageal diseases with promising sensitivity and specificity. Further prospective evaluation of the classifier in the early detection of ESCAs in high-risk individuals is warranted.

**Supplementary Information:**

The online version contains supplementary material available at 10.1186/s12916-021-02109-y.

## Background

Esophageal cancer (ESCA) is one of the most deadly cancers with poor prognosis and increasing incidence worldwide [1]. Due to the absence of specific symptoms, approximately 40% ESCA patients have advanced disease at diagnosis [2] and the 5-year survival rate for those patients is less than 5% [2]. Thus, detecting early-stage ESCA when curable treatments are possible is a pivotal way to prolong survival. Even though endoscopy has been recommended to high-risk individuals for the early detection of ESCA in geographic regions with high prevalence [3, 4], it is not suitable for large-scale screening due to its invasive, inconvenient, and time-consuming process [5]. Therefore, the development of a noninvasive or minimally invasive method for ESCA early detection is imperative in the clinic.

Circulating cell-free DNA (cfDNA)-based liquid biopsy has shown potential to revolutionize the early detection of cancers by enabling minimally invasive molecular testing of solid tumors [6]. Genetic aberrations such as mutations, small insertions and deletions, copy number variations, and epigenetic alterations shed by tumors can be detected in cfDNA using next-generation sequencing (NGS) [7]. Among these, cfDNA methylation stands out in the early detection of cancers due to its early occurrence during tumorigenesis and rich signal abundance for analysis [6]. There are nearly 30 million methylation sites across the human genome, making them a ubiquitous and rich signal to detect cancer even with a low concentration of cfDNA [8].

The cfDNA methylation has been studied in the early detection of multiple cancers including ESCA [9–12]. A gene panel with 5 methylation differential markers (MDMs) sequenced by quantitative methylation-specific polymerase chain reaction (PCR) could discriminate ESCA from healthy controls, with a specificity of 91% and sensitivity of 43%, 64%, 77%, and 92% for stage I–IV, respectively [12]. Another study has shown that a ctDNA methylation classifier could separate ESCA patients from healthy individuals with sensitivity of 0–20% for patients with stage I and 60–75% for patients with stage II [11]. However, the performance of cfDNA methylation in the early detection of ESCA is far from satisfactory and can be improved with well-designed clinical trials.

In this study, we aimed to identify ESCA-specific differentially methylated regions (DMRs) and evaluate the potential performance of cfDNA methylation markers in the early detection of ESCA through four well-designed stages: panel design, marker selection, model development, and model validation. We first compared the methylation profiles between ESCA tumor and paired adjacent tissues from the Cancer Genome Atlas (TCGA) and in-house data and identified ESCA-specific DMRs. We then built and tested a cfDNA methylation classifier using a support vector machine (SVM)-based machine learning to differentiate ESCA from healthy controls and benign esophageal diseases. At last, the diagnostic performance of the early detection classifier was validated in an independent validation cohort.

## Materials and methods

### Study design and participants

**Fig. 1:**
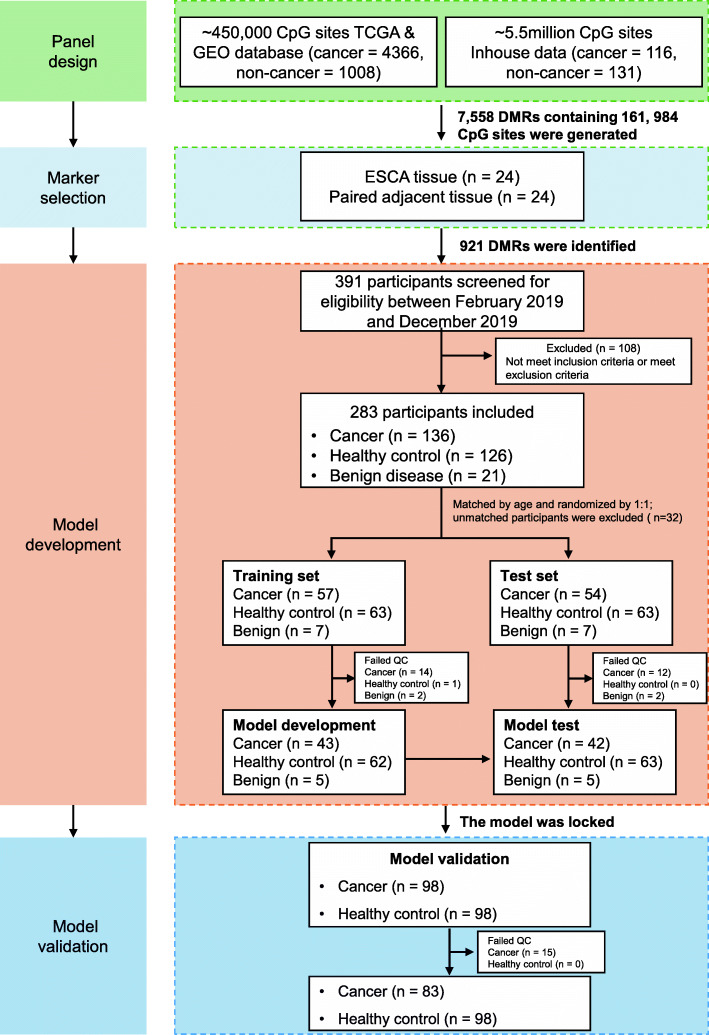
Flow diagram of the study

This is a multicenter, case-control study including four stages: (1) panel design, (2) marker selection, (3) model development, and (4) model validation (Fig. 1).

#### Panel design

As previously described [13], public data sets including TCGA and GEO databases (tumor = 4366, normal = 1008; HumanMethylation450K array) and in-house generated functional methylome (targeted methylation panel, 5.5 million CpG sites) sequencing data (tumor = 116, normal = 131) were used in the present study. The methylation data of TCGA datasets (https://portal.gdc.cancer.gov/) was analyzed by limma (R package) along with the in-house data to select differentially methylated CpG sites (Benjamini–Hochberg-corrected FDR < 0.05). The methylation data of the GEO dataset with 656 normal WBC samples under the accession code GSE40279 [14] was used to remove hypermethylated CpG sites in the hematopoietic lineage (> 0.1). CpG sites that were located on X or Y chromosomes were also excluded. In addition, CpG sites that were reported to be associated with common cancers were also included. Altogether, this yielded a total of 161,984 CpG sites in the panel design phase, spanning ~2.7Mb of the human genome in six common cancer types including ovarian, lung, colorectal, pancreatic, liver, and esophageal cancers. The panel was originally developed for early detection and tissue-of-origin of multi-type tumors.

#### Marker selection for ESCA

Tumor and paired adjacent tissues of ESCA patients from Guangdong Provincial People’s Hospital were collected and profiled with the above target methylation panel. Esophageal tumors and paired adjacent tissues were sampled in treatment-naive patients through esophagectomy. All formalin-fixed and paraffin-embedded (FFPE) tissues went through a second research histopathology review by an independent expert pathologist before DNA extraction. Tumor tissues that contained less than 30% cancer cells or failed to meet the DNA quality control (QC) criterion were excluded from the subsequent analysis. The CpG sites/loci were grouped into DMRs based on the co-methylation levels and genomic distances of adjacent CpG sites (detailed definition of DMR see the “ELSA-seq” section in the “Materials and methods” section, Additional file 1: Figure S1A). ESCA-specific DMRs were selected using a modified Wald test with an adjusted *P*-value < 0.05 and an absolute mean difference ≥ 0.2.

#### Model development

Blood samples from patients with pathologically diagnosed ESCA and benign esophageal diseases were obtained from February 2019 to December 2019 in Guangdong Provincial People’s Hospital.

Inclusion criteria: (1) 40–75 years old and able to provide written informed consent. (2) The diagnosis of esophageal cancer (stage I–IV or high-grade dysplasia/carcinoma in situ [stage 0]) or benign esophageal diseases could be confirmed within 90 days prior to blood collection, based on the assessment of a pathological specimen. Benign esophageal diseases enrolled in this study included but not limited to Barrett’s esophagus, heterotopic gastric mucosa (HGME), and leiomyoma of the esophagus, or (3) a high suspicion of esophageal cancer or esophageal benign disease by clinical and/or radiological assessment, with planned biopsy or surgical resection to confirm diagnosis within 4 weeks (28 days) after study blood draw. (4) Plasma samples could be collected prior to any treatment including local/regional therapy, surgery, radiation, or systemic chemotherapy. Pathological stages of all patients were determined by the researchers based on the 8th edition of the American Joint Committee on Cancer (AJCC) classifications [15].

Exclusion criteria: (1) Participants who were ever diagnosed with any other cancer. (2) Participants who had received antibiotic therapy within 14 days prior to blood draw. (3) Participants who had received blood transfusions or blood products within 30 days prior to blood draw. (5) Participants who were currently taking any antiplatelet or anticoagulant therapies. (6) Participants who had received organ transplantation or allogeneic hematopoietic stem cell transplantation. (7) Participants who could not tolerate blood draw.

Healthy controls were recruited from Chongqing University Cancer Hospital from September 2019 to December 2019, defining as participants who were free from history of malignancy, as well as critical illness including hepatitis, liver cirrhosis, chronic obstructive pulmonary disease, and colorectal disease. All healthy volunteers would receive routine healthy checkups including routine blood test, urinalysis, blood biochemical tests, electrocardiograms, thoracic low-dose computer tomography (CT), and abdominal ultrasound. Participants with normal test results would be included in the study. All participants were aged 40–75 years. After being matched by age, ESCA, benign esophageal disease and healthy controls were randomized by 1:1 into the training and test cohort. Blood samples that failed DNA QC criterion were excluded from the downstream analysis.

#### Model validation

Blood samples from patients with ESCA in the First Affiliated Hospital of Zhengzhou University were obtained from February 2020 to May 2020 to further validate the performance of the early detection classifier independently. Healthy controls were also recruited from Chongqing University Cancer Hospital from February 2020 to May 2020. The inclusion and exclusion criteria were the same as the above. The methylation classifier developed from the training and test cohort was locked before the independent validation cohort was recruited. For the independent validation set, the clinical information (e.g., cancer or healthy status) was blinded to the researchers who performed sequencing, quality control, and classification analyses.

This study was approved by the Ethics Committees of Guangdong Provincial People’s Hospital, Chongqing University Cancer Hospital, and First Affiliated Hospital of Zhengzhou University (GDREC2019687H; 2019-KY-394). All participants provided informed consents.

### ELSA-Seq

All sequencing experiments were implemented in a College of American Pathologists (CAP)- and Clinical Laboratory Improvement Amendments (CLIA)-certified laboratory (Burning Rock Biotech, Guangzhou, China). Deep targeted bisulfite sequencing (ELSA-seq) was performed on tissue samples with an average sequencing depth of 500× and plasma samples with an average sequencing depth of 1000× [16].

The procedures for DNA extraction were as previously described [17]. In brief, for tissue samples, DNA was extracted with a QIAamp DNA formalin-fixed and paraffin-embedded (FFPE) tissue kit according to the manufacturer’s instructions. DNA concentration was measured by the Qubit double-stranded DNA assay (Life Technologies, Carlsbad, CA). For blood samples, 8–10 ml of whole blood samples for each participant were collected by Streck Cell-Free DNA BCT® (Streck, USA) and centrifuged at 1600 g for 20 min at room temperatures to obtain the plasma. All plasma was stored at −80 °C. The QIAamp Circulating Nucleic Acid Kit (551114, Qiagen, Valencia, CA, USA) was used to extract cfDNA from plasma.

As for methylation sequencing, a capture-based method was used to detect CpG sites. The bisulfite sequencing library was generated via the brELSATM method [18] (Burning Rock Biotech, Guangzhou, China). The target libraries were quantified by real-time PCR and sequenced on NovaSeq 6000 with 1000× target depth on average. With the raw sequencing data, several bioinformatics tools including Trimmomatic, BWA-meth, and samblaster were applied to the alignment and caller of reads as the downstream analysis. About 60–80% of reads uniquely aligned on the bait regions (target ratio) and more than 90% of bait regions covered by over 300 reads (uniformity) with 10–30 ng input cfDNA. For each CpG site, the median effective coverage depth was 329×. Since differentially methylated region consisting of multiple CpG sites played more important roles than a single CpG site in cancer detection as reported [19], we defined CpG sites with close genomic distance and highly correlation in methylation level as DMRs (Additional file 1: Figure S1A). In total, 7558 DMRs were generated based on the 161,984 CpG sites.

The score for each DMR was calculated according to both depth of coverage and the distance between the adjacent CpG sites as follows [13]:

$$ \mathrm{Methylation}\ \mathrm{Region}\ \mathrm{Score}=\frac{1}{n}\times {\sum}_{i=1}^n\ \left(\frac{\sum_{j=1}^m{l}_{ij}^2}{L_i^2}\right) $$

In brief, for a given methylation region, *n* is the total number of reads that cover several CpG sites, and *L*_*i*_ is the number of CpG sites covered on *i*th read. *l*_*ij*_ is denoted as the length of successive methylated CpG sites (>1), and *m* is the total counts on *i*th read. The number of reads in each region was used to normalize the depth difference, bounding the metric between 0 and 1.

### Machine learning algorithm for model development

Supporting vector machine (SVM) algorithm [20] was implemented to build the early detection classifier to distinguish cancers from benign/healthy samples by Scikit-learn (version 0.20.4) [21]. Fivefold cross-validation was used to test the performance of the SVM classifier as well as to find the optimal regularization parameter C of the classifier. Specifically, within five equally sized folds, each fold of the training samples was used as the test group once, while the rest four folds of samples containing both case and control samples were used to build the model and to further predict the “label” of each test group sample. In each cross-validation fold, the sample size ratio between case and control was set comparable. Overall, all samples in the cross-validation group obtained an independent prediction result, and sensitivity and specificity were calculated.

### Statistical analysis

For the training and test sets respectively, assuming the AUC of 0.93, it was estimated that a minimum of 44 cases and 44 controls respectively would provide 90% power to distinguish an estimated two-sided test of significance set at the 5% level with a null value of 0.8. The independent validation set was designed to have a power of 80% to test the pre-specified hypothesis that the classifier would have a sensitivity of 55% or more for the detection of ESCA at one-sided type I error of 5%. It required at least 70 patients with ESCA.

Continuous variables were described with mean ± SD and were compared by a 2-sided *t* test or the Mann-Whitney *U* test. Categorical variables were described with number (percentages) and compared by chi-square test or Fisher’s exact test. Gene Ontology (GO) enrichment analysis of the genes containing ESCA-specific DMRs was performed using DAVID (Database for Annotation, Visualization and Integrated Discovery) [22]. Fivefold cross-validation was applied in the training dataset, and a supporting vector machine was used to build a two-category classifier to distinguish cases and controls. The area under the curve (AUC) and 95% confidence interval (CI) were generated to evaluate the model performance. The cutoff value for the early detection model was determined by Youden’s index. The 95% CIs for sensitivity and specificity were generated using the Clopper-Pearson method [23, 24]. Comparisons between AUCs were performed using the DeLong method [25, 26]. A two-sided *P* value of 0.05 was set as the level of significance. The statistical analyses were performed using R 3.4.2 and MedCalc v19.3.1.

## Results

### Methylation marker refinement

We previously designed a targeted methylation panel for the early detection and tissue-of-origin of multiple cancers including lung, colorectal, ovarian, pancreatic, and esophageal cancers [27]. To improve the performance of this targeted methylation panel in the early detection of ESCA, we first optimize the pre-designed panel by performing methylation targeted sequencing in 24 treatment-naïve ESCA tumor and matched adjacent tissue samples. The baseline characteristics for the 24 ESCA patients are shown in Table 1. In detail, the majority of them were male (75%), non-smokers (75%), and non-drinkers (100%). Most (96%) patients were more than 55 years old and most (92%) were esophageal squamous cell cancer (ESCC). The numbers of stages I–IV were 4 (17%), 13 (54%), 4 (17%), and 2 (8%), respectively.

**Table 1:** The characteristics of participants

Parameters	Marker selection	Training set			Test set			Independent validation set	
	Cancer (*n* = 24)	Cancer (*n* = 43)	Control (*n* = 67)	*P* value	Cancer (*n* = 42)	Control (*n* = 68)	*P* value	Cancer (*n* = 83)	Control (*n* = 98)
Age, *n* (%)				0.29			0.22		
≥ 55	23 (96%)	33 (77%)	44 (66%)		31 (74%)	41 (60%)		77 (92.8%)	57 (58.2%)
40–55	1 (4%)	10 (23%)	23 (34%)		11 (26%)	27 (40%)		6 (7.2%)	41 (41.8%)
Sex, *n* (%)				0.002			1.00		
Female	6 (25%)	7 (16%)	31 (46%)		14 (33%)	23 (34%)		32 (38.6%)	52 (53.1%)
Male	18 (75%)	36 (84%)	36 (54%)		28 (67%)	45 (66%)		51 (61.4%)	46 (46.9%)
Smoking history, *n* (%)				0.02			0.44		
Smokers	6 (25%)	12 (28%)	7 (10%)		9 (21%)	10 (15%)		24 (28.9%)	39 (39.8%)
Non-smokers	18 (75%)	31 (72%)	60 (90%)		33 (79%)	58 (85%)		52 (62.7%)	59 (60.2%)
Unknown								7 (8.4%)	
Alcohol history, *n* (%)				1.00			0.10		
Drinkers	0	4 (9%)	7 (10%)		3 (7%)	13 (19%)		14 (16.9%)	48 (49.0%)
Non-drinkers	24 (100%)	39 (91%)	60 (90%)		39 (93%)	55 (81%)		59 (71.1%)	50 (51.0%)
Unknown								10 (12.0%)	
Pathological type									
ESCC, *n* (%)	22 (92%)	39 (91%)			38 (90%)			72 (86.7%)	
EAC, *n* (%)	2 (8%)	4 (9%)			4 (10%)			8 (9.6%)	
Others, *n* (%)								3 (3.6%)	
Stage, *n* (%)									
Stage 0	4 (17%)	3 (7%)			2 (5%)			10 (12.0%)	
Stage I	13 (54%)	9 (21%)			8 (19%)			14 (16.9%)	
Stage II	4 (17%)	11 (26%)			13 (31%)			27 (32.5%)	
Stage III	2 (8%)	12 (28%)			7 (17%)			14 (16.9%)	
Stage IV	1 (4%)	8 (19%)			6 (14%)			18 (21.7%)	
Unknown									
Healthy control, *n* (%)			62 (93%)			63 (93%)			98 (100%)
Benign disease, *n* (%)			5 (7%)			5 (7%)			

**Fig. 2:**
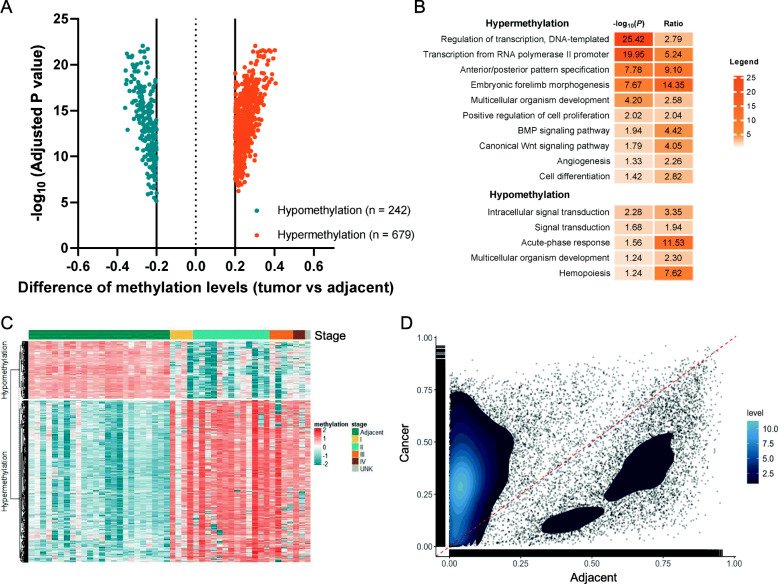
Methylation marker selection. **A** Significant difference of methylation levels between ESCA tissues and paired adjacent tissues. **B** Gene ontology enrichment analyses of the genes containing significantly hypomethylated or hypermethylated MDRs. **C** Heatmap illustrating the hypomethylated and the hypermethylated DMRs between ESCA tissues and adjacent tissues. **D** Scatter diagram exhibiting the distribution of methylation region value between the ESCA tissues and paired adjacent tissues. Abbreviations: DMR, differentially methylated region; ESCA, esophageal cancer

By comparing the methylation signatures between tumor and paired adjacent tissues, we identified 921 ESCA-specific DMRs with the highest statistical significance and absolute mean difference ≥ 0.2 between ESCA tumor and adjacent tissues (Fig. 2A). The median region size of those DMRs was approximately 228 bp and there were around 19 CpG sites per region on average. Of these 921 DMRs, 679 (73.7%) showed a higher methylation level in the tumor tissues, while the rest (26.3%) showed a lower methylation level in the tumor tissues (Fig. 2A). Genes that contained those DMRs were generated by the annotatePeaks function (software Homer). There were 340 genes involved in the hypermethylated DMRs, enriching in the pathways involved in the regulation of transcription (transcription from RNA polymerase II promoter), cellular fate (positive regulation of cell proliferation; cell differentiation), organism development (anterior/posterior pattern specification; multicellular organism development), and tumorigenesis (BMP signaling pathway; canonical Wnt signaling pathway) via GO enrichment analysis, while there were 219 genes involved in the hypomethylated DMRs, enriching in the pathways related to intracellular signal transduction, acute-phase response, multicellular organism development, and hemopoiesis (Fig. 2B). Several signaling pathways are related with oncogenesis and regulation of oncogenes or tumor suppressor genes, indicating the biological rationality for the methylation marker selection. As shown in the Sankey plot (Additional file 1: Figure S1B), the ESCA-specific DMRs exhibited a higher proportion of hypermethylation in CpG islands and a higher proportion of hypomethylation in CpG shores, CpG shelves, and open sea regions, and most of them were related with protein-coding function.

The methylation levels for the 921 methylation regions are depicted in Fig. 2C, showing the different methylation patterns between tumors and paired adjacent tissues. The regions of the methylation level of ESCA tumor and adjacent tissues were significantly enriched in both sides of the diagonal line (Fig. 2D). Moreover, the robust differentiation was further validated by unsupervised clustering based on the tumor and paired adjacent tissues, showing the similar methylation patterns within tumors instead of adjacent tissues (Additional file 1: Figure S1C). The methylation levels for each CpG site involved in the 921 DMRs are also depicted in Additional file 1: Figure S1D, showing the different methylation levels between tumor and adjacent tissues. The principal component analysis further demonstrated the distinct component between tumor and adjacent tissues (Additional file 1: Figure 1E). All together, these results indicated the robust discrimination between cancer and adjacent tissue based on the selected methylation biomarkers.

### Early detection model development

We further explored whether the 921 DMRs identified in ESCA tissues would differentiate patients with ESCA from healthy controls and benign esophageal disease through cfDNA sequencing. Participants with ESCA and benign esophageal disease and healthy controls were matched by age and randomized into the training and test sets. After samples that failed quality control were excluded, 110 participants (43 cancer, 62 healthy control, and 5 benign esophageal disease) were included in the training set and 110 participants (42 cancer, 63 healthy control, and 5 benign esophageal disease) were in the test set. The detailed characteristics of cases and controls in training and test sets are also demonstrated in Table 1. The age was relatively balanced between cases and controls in the training (*P* = 0.29) and test sets (*P* = 0.22). The tumor stages were similar between training and test sets. However, there were more smokers and males in the ESCA group than in the control group in the training set (*P* < 0.05).

**Fig. 3:**
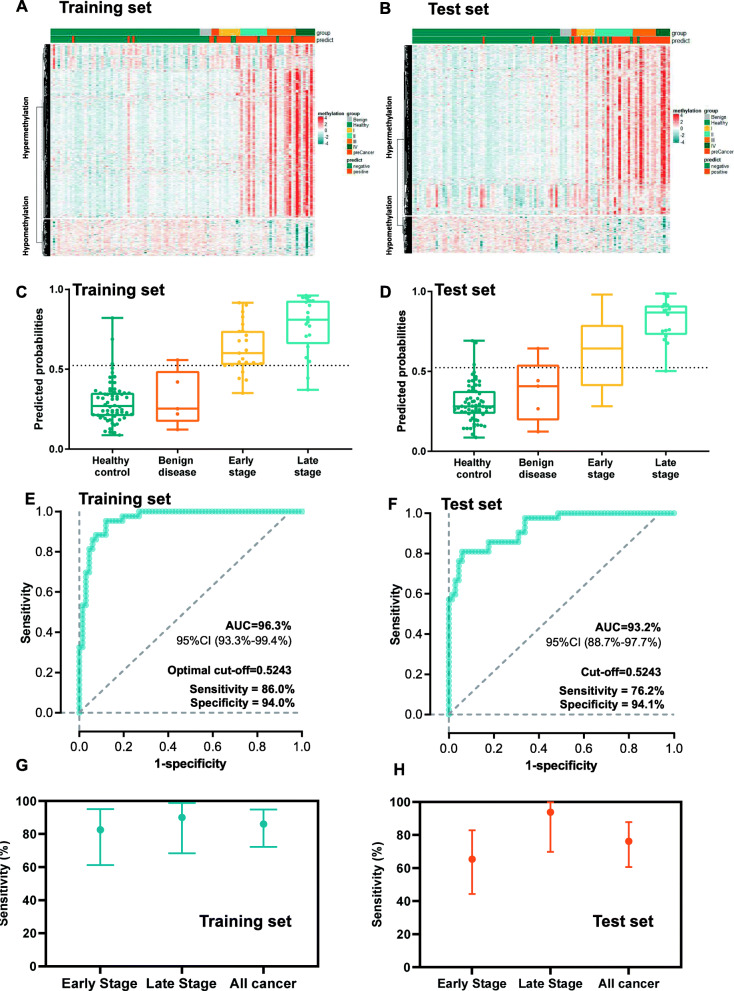
Early detection model development. **A**, **B** Heatmap illustrating the cfDNA methylation levels for the selected DMRs between participants with ESCA, benign esophageal diseases, or healthy controls in the training (**A**) and test (**B**) sets. **C**, **D** Predicted probabilities of healthy control, benign esophageal diseases, and ESCA with different clinical stages in the training (**C**) and test (**D**) sets. **E**, **F** Receiver operating characteristic curve delineating the association between predictive probability and cancer in the training (**E**) and test (**F**) sets. **G**, **H** Sensitivity for ESCA with early and late stages in the training (**G**) and test (**H**) sets. Abbreviations: DMR, differentially methylated region; ESCA, esophageal cancer

The cfDNA methylation levels for the selected 921 ESCA-specific DMRs in the training and test sets are depicted in Fig. 3A and B, showing the different cfDNA methylation patterns between ESCA and controls. Based on the DMRs identified from the tissue samples, a supervised machine learning model was implemented and cross-validation was used in the training set to classify the DNA methylation profile of blood samples as tumor and non-tumor. Fivefold cross-validation yielded high accuracy with a mean area under the curve of 0.96 (Additional file 1: Figure S2). The predicted probabilities were increased with tumor stage and were significantly higher in cancers than those in healthy controls and benign esophageal diseases in both the training and test sets (*P* < 0.05, Fig. 3C, D). Using the best cutoff value, as determined via Youden’s index, the methylation markers demonstrated sensitivity and specificity of 86.0% (95% CI, 72.2−94.8%) and 94.0% (95% CI, 85.5−98.3%), respectively, for discriminating ESCA from healthy controls and benign esophageal disease in the training dataset, and 76.2% (95% CI, 60.5–87.9%) and 94.1% (95% CI, 85.7−98.4%) in the test dataset (Table 2), yielding AUCs of 0.963 (95% CI, 0.933−0.994) and 0.932 (95% CI, 0.887−0.977) in the training and test datasets, respectively (Fig. 2E, F). Sensitivity increased with tumor stages as demonstrated in Additional file 1: Table S1 with sensitivity of 82.6% (95% CI, 61.2−95.0%) and 90.0% (95% CI, 68.3−98.8%) for early-stage (stage 0–II) and late-stage (stage III–IV) patients in the training set and 65.4% (95% CI, 44.3−82.8%) and 93.8% (95% CI, 69.8−99.8%) for early-stage and late-stage patients in the test set, respectively (Fig. 2G, H, Table 2).

**Table 2:** Sensitivity and specificity in the training set and test set

Patient group	Training set				Test set			
	Tested	Positive	Sensitivity (%)	Specificity (%)	Tested	Positive	Sensitivity (%)	Specificity (%)
Early stage	23	19	82.6% (61.2–95.0%)		26	17	65.4% (44.3–82.8%)	
Late stage	20	18	90.0% (68.3–98.8%)		16	15	93.8% (69.8–99.8%)	
All cancer	43	37	86.0% (72.2–94.8%)		42	32	76.2% (60.5–87.9%)	
Benign	5	1	20.0% (0.5–71.4%)	80.0% (28.4–99.5%)	5	1	20.0% (0.5–71.4%)	80.0% (28.4–99.5%)
Healthy control	62	3		95.2% (86.5–99.0%)	63	3		95.2% (86.7–99.0%)
All non-cancer	67	4		94.0% (85.5–98.3%)	68	4		94.1% (85.7–98.4%)

In the total population including training and test sets, specificity was 95.2% for healthy individuals and 80% for benign esophageal diseases, and sensitivity was 60%, 77.8%, 86.4%, and 100.0% for patients with stage 0–IV, respectively (Additional file 1: Figure S3A). In total, these results suggested that the cfDNA methylation classifier might effectively differentiate ESCA from healthy control and benign esophageal disease.

To further examine whether the performance of the classifier was influenced by clinical characteristics, we performed subgroup analysis by stratifying the total participants by age, sex, and pathological diagnosis. No significant difference of the performance of methylation classifier was observed (Additional file 1: Table S2), and the predicted probabilities of ESCA patients stratified by those clinical covariates were still higher than those in healthy controls (*P* < 0.05, Additional file 1: Figure S3B), indicating that the performance of our cfDNA methylation classifier was stable and not influenced by those clinical characteristics.

### Independent validation of the early detection classifier

To further validate the performance of the established methylation classifier, we prospectively enrolled an independent plasma validation cohort including participants with ESCA and healthy controls. The detailed characteristics of cases and controls are also provided in Table 1. In brief, most of the patients included were 55 years or older (98.2%) and had a high proportion of ESCC (86.7%), which was consistent with the training set and test set. Moreover, 61.4% patients were stage 0–II.

**Fig. 4:**
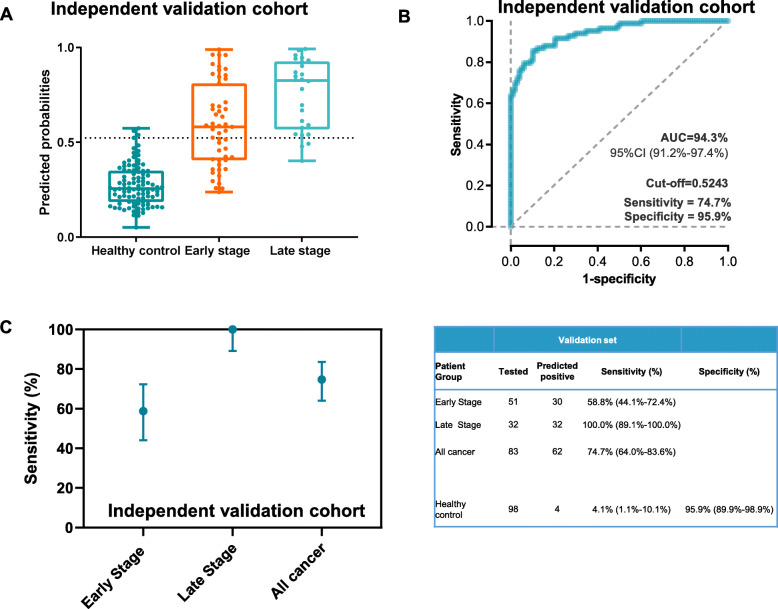
Independent validation of the early detection classifier. **A** Predicted probabilities of healthy control, benign diseases, and ESCA with different clinical stages in the independent validation set. **B** Receiver operating characteristic curve delineating the association between predictive probability and cancer in the independent validation cohort. **C** Sensitivity and specificity of ESCA with early and late stages in the independent validation cohort

The predicted probabilities were also increased with tumor stage and were significantly higher in patients with ESCA than healthy controls (*P* < 0.05, Fig. 4A). In addition, the cfDNA methylation classifier with the above cutoff value had sensitivity and specificity of 74.7% (64.0–83.6%) and 95.9% (89.9–98.9%), respectively, to discriminate ESCA from normal controls, yielding AUCs of 0.943 (95% CI, 0.912−0.974, Fig. 4B). Similarly, sensitivity also increased with tumor stages as demonstrated with sensitivity of 58.8% (95% CI, 44.1−72.4%) and 100.0% (95% CI, 89.1−100.0%) for early-stage and late-stage patients (Fig. 4C, Additional file 1: Table S3), suggesting the promising utility of the cfDNA methylation in the detection of early-stage ESCA.

We also performed subgroup analysis in the test set. Similarly, the predicted probabilities of ESCA patients stratified by the clinical variables, such as age, smoking status, and drinking history, were still higher than those in healthy controls (*P* < 0.05, Additional file 1: Figure S4A). Altogether, our results further confirmed the robust performance of the cfDNA classifier to differentiate ESCA from healthy controls.

## Discussion

Currently, no standard screening approach is recommended for ESCA in the general population. Endoscopy is the gold standard for the diagnosis of ESCA; however, its invasiveness and inconvenience limit the clinical utility in ESCA screening. Hence, the development of cfDNA-based early detection technology would be transformative. In the present study, we first identified 921 ESCA-specific DMRs by comparing ESCA tumor and paired adjacent tissues. An early detection cfDNA classifier was first built and tested based on the selected ESCA-specific DMRs and further validated in another independent prospective plasma validation cohort with a sensitivity of 74.7% and a specificity of 95.9%. Altogether, our results demonstrated that cfDNA-based methylation was a promising approach in the early detection of ESCA.

The development of the early detection classifier has gone through thorough refinement and validation. The panel was originally developed for the early detection of multiple cancers. To optimize the analytic performance in ESCAs, we first refined the methylation markers and identified the ESCA-specific DMRs. Previous cfDNA methylation studies mainly focused on the DMRs derived from cancer and non-cancer in the western population [12]. However, the eastern population presented significant geographic and ethnic variations [28] and the difference between ethnicities should not be overlooked. The specific DMRs based on the Chinese population for ESCA early detection are imperative. The methylation markers were further selected based on 24 paired tissues between ESCAs and adjacent samples, and most of these markers were involved in the regulation of transcription, cell proliferation and differentiation, intracellular signaling transduction, and regulation of tumor, demonstrating the biology feasibility for a noninvasive plasma assay for detection of esophageal cancer. We then trained, tested, and independently validated the classifier to demonstrate the robust performance of the classifier to differentiate patients with ESCA in plasma samples. To be mentioned, the independent validation cohort was enrolled after the model was locked, and clinical information was blinded to the analysts who performed sequencing, and classification analyses to reduce potential bias.

The performance of cfDNA methylation in the early detection of ESCA has been studied in a few studies. In the Circulating Cell-free Genome Atlas (CCGA) study, cfDNA methylation performed well in multi-cancer detection with a sensitivity of 43.9% in stage I–III at a specificity of 99.3%. However, the sensitivity was 0−20% for stage I in ESCA. Another recent study demonstrated a diagnostic performance for stage I ESCA with a sensitivity of 43% at a specificity of 91% [12]. The performance of cfDNA methylation was far from satisfactory in the detection of ESCA, especially in the early stage of ESCA. In the present study, 15 patients with stage 0 ESCA and 31 patients with stage I ESCA were included, yielding sensitivity of 50% and 62.5% in the test cohort and 40% and 35.7% in the independent validation cohort, respectively. Since patients with early stage or carcinoma in situ would have better prognosis than those with late stage, the identification of more patients with early stage or carcinoma in situ would provide more clinical significance.

Several benign esophageal diseases were also included in the present study, though with a relatively small sample size. The current methylation model yielded a specificity of 80% for benign esophageal disease in both training and test sets (*n* = 10). We reviewed the pathological diagnosis for these patients, and one patient with esophagitis and another one with HGME were identified as positive, which needed to be further examined by esophagoscopy. However, the diagnostic performance for benign esophageal diseases needs to be further validated in a larger population.

It is also worth noting that our study is limited by including relatively small sizes of patients with esophageal adenocarcinoma (EAC) due to the geographic characteristics. Despite that ESCC and EAC are biologically distinct cancers, the DMRs identified in this study shared significant overlap signatures in EAC and ESCC. These similar methylation signatures suggested these candidate DMRs were representative for both pathological subtypes. In addition, to test whether the model performance was confounded by pathological type and other clinical covariates, we further compared the AUCs stratified by these clinical covariates, such as sex, age, and histology subtypes, and no significant differences were observed, indicating the robust discrimination for our methylation classifier is not influenced by clinical confounding factors.

However, limitations should not be overlooked in the present study. Firstly, the nature of the present study was a case-control study, even though three sets were used. The performance of the classifier in asymptomatic high-risk individuals needs to be further studied. Secondly, the ESCA patients included in the present study were individuals with known cancers, most of whom were diagnosed because of symptoms, which may overestimate the performance of the classifier in real-world where there are more early-stage patients in asymptomatic and screened individuals. Thirdly, the number of participants with EAC and benign esophageal disease was relatively low. The ability to detect EAC or differentiate benign esophageal disease needs to be improved by including more participants in the future. However, by comparing the AUCs in the subgroup analysis, we did not observe a significant difference in the performance of the classifier between ESCC and EAC.

## Conclusion

This study demonstrates that the cfDNA methylation classifier is promising for the early detection of ESCAs. We anticipate that noninvasive cfDNA methylation will have an increasingly important role in cancer screening in the future.

## Supplementary Information

**Additional file 1.**

## Data Availability

All relevant data supporting this study’s key findings are available within the supplementary information files and available from the corresponding author upon reasonable request.

## Abbreviations

AJCC: American Joint Committee on Cancer
AUC: Area under the curve
CAP: College of American Pathologists
CCGA: Circulating Cell-free Genome Atlas
cfDNA: Cell-free DNA
CI: Confidence interval
CLIA: Clinical Laboratory Improvement Amendments
CT: Computer tomography
DMRs: Differentially methylated regions
EAC: Esophageal adenocarcinoma
ELSA-seq: Deep targeted bisulfite sequencing
ESCA: Esophageal cancer
ESCC: Esophageal squamous cell cancer
FFPE: Formalin-fixed and paraffin-embedded
GO: Gene Ontology
HGME: Heterotopic gastric mucosa
MDMs: Methylation differential markers
NGS: Next-generation sequencing
PCR: Polymerase chain reaction
QC: Quality control
SVM: Support vector machine
TCGA: The Cancer Genome Atlas
