# Supplementary material for: Discovery and validation of methylation signatures in circulating cell-free DNA for early detection of esophageal cancer: a case-control study
Source: BMC Med. 2021 Oct 13;19:243. doi: 10.1186/s12916-021-02109-y (PMC8513367; doi:10.1186/s12916-021-02109-y)
Supplement: Supplementary file 1 — Additional file 1. [file 12916_2021_2109_MOESM1_ESM.docx]

**Online Additional File**

G Qiao, W Zhuang, B Dong et al. Discovery and validation of methylation signatures in circulating cell-free DNA for early detection of esophageal cancer: a case-control study

**Contents:**

**Additional File Tables: 3**

**Additional File Figure: 1-4**

**Table S1. Sensitivity and specificity in training set and test set.**

| **Patient Group** | **Training set** | | | |  | **Test set** | | | |
| --- | --- | --- | --- | --- | --- | --- | --- | --- | --- |
|  | **Tested** | **Positive** | **Sensitivity (%)** | **Specificity (%)** |  | **Tested** | **Positive** | **Sensitivity (%)** | **Specificity (%)** |
| Stage 0 | 3 | 2 | 66.7% (9.4%-99.2%) |  |  | 2 | 1 | 50.0% (1.3%-98.7%) |  |
| Stage I | 9 | 7 | 77.8% (39.8%-97.2%) |  |  | 8 | 5 | 62.5% (24.5%-91.5%) |  |
| Stage II | 11 | 10 | 90.9% (58.7%-99.8%) |  |  | 16 | 11 | 68.8% (41.3%-89.0%) |  |
| Stage III | 12 | 10 | 83.3% (51.7%-97.9%) |  |  | 10 | 9 | 90.0% (55.4%-99.8%) |  |
| Stage IV | 8 | 8 | 100.0% (63.1%-100.0%) |  |  | 6 | 6 | 100.0% (54.1%-100.0%) |  |
| All cancer | 43 | 37 | 86.0% (72.2%-94.8%) |  |  | 42 | 32 | 76.2% (60.5%-87.9%) |  |
|  |  |  |  |  |  |  |  |  |  |
| Benign | 5 | 1 | 20.0% (0.5%-71.4%) | 80.0% (28.4%-99.5%) |  | 5 | 1 | 20.0% (0.5%-71.4%) | 80.0% (28.4%-99.5%) |
| Healthy control | 62 | 3 |  | 95.2% (86.5%-99.0%) |  | 63 | 3 |  | 95.2% (86.7%-99.0%) |
| All non-cancer | 67 | 4 |  | 94.0% (85.5%-98.3%) |  | 68 | 4 |  | 94.1% (85.7%-98.4%) |

**Table S2. AUCs stratified by clinical covariates**

|  | Yes | No | P |
| --- | --- | --- | --- |
| Male | 0.968 (0.944-0.991) | 0.902 (0.832-0.972) | 0.08 |
| Age (≥55） | 0.941 (0.901-0.977) | 0.953 (0.907-1.000) | 0.68 |
| ESCC | 0.951 (0.925-0.978) | 0.903 (0.803-1.000) | 0.33 |

ESCC, Esophageal squamous cell carcinoma; AUC, Area under roc curve.

**Table S3. Sensitivity and specificity in the validation set.**

| **Patient Group** | **Validation set** | | | |
| --- | --- | --- | --- | --- |
|  | **Tested** | **Positive** | **Sensitivity (%)** | **Specificity (%)** |
| Stage 0 | 10 | 4 | 40.0% (12.2%-73.8%) |  |
| Stage I | 14 | 5 | 35.7% (12.8%-64.9%) |  |
| Stage II | 27 | 21 | 77.8% (57.8%-91.4%) |  |
| Stage III | 14 | 14 | 100.0% (76.8%-100.0%) |  |
| Stage IV | 18 | 18 | 100.0% (81.5%-100.0%) |  |
| All cancer | 83 | 62 | 74.7% (64.0%-83.6%) |  |
|  |  |  |  |  |
| Healthy control | 98 | 4 |  | 95.9% (89.9%-98.9%) |


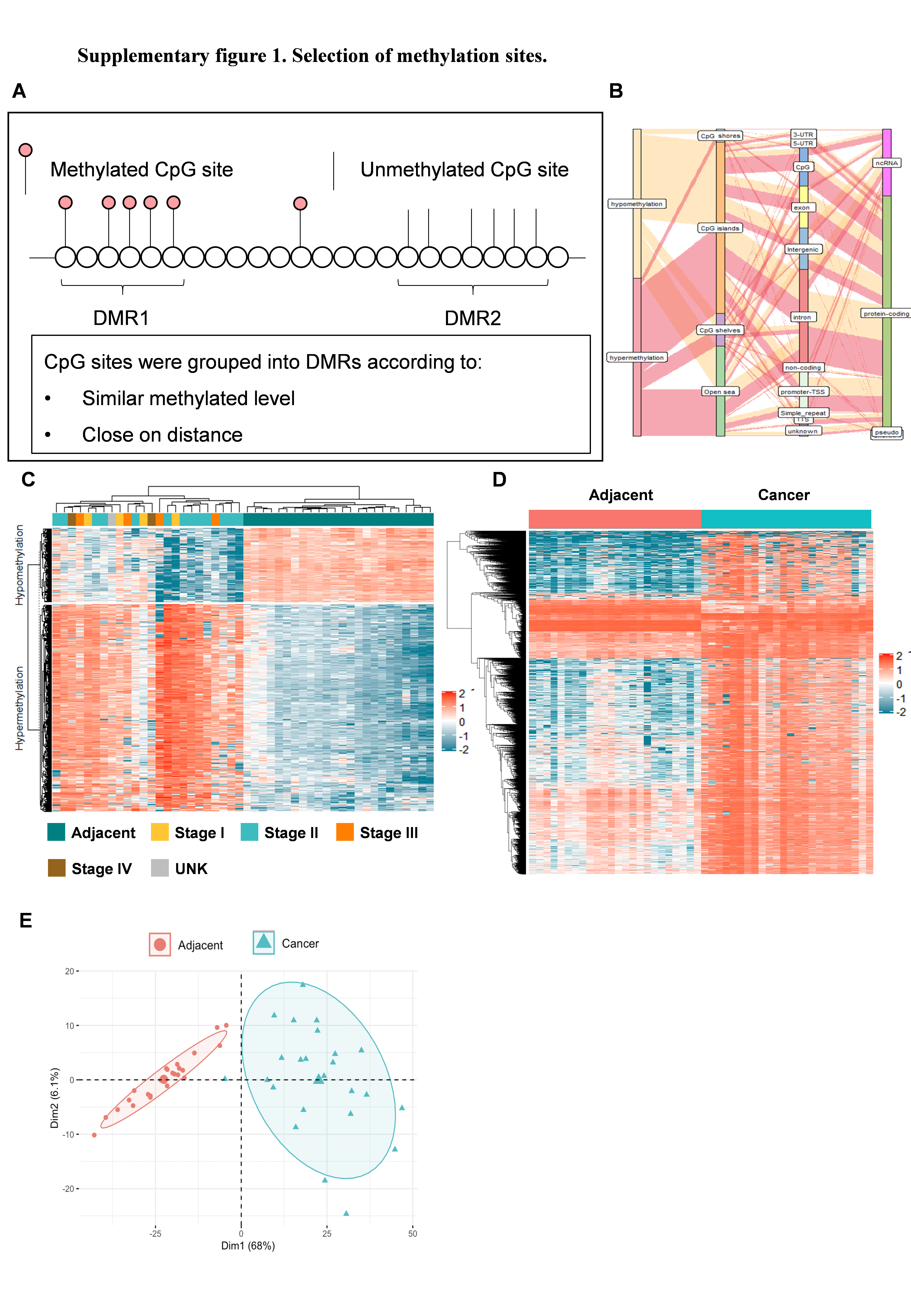


**Figure S1. Selected methylation sites.**

**(A).** Definition of DMRs. **(B).** Sankey plot of the ESCA -specific DMRs. **(C).** Heatmap illustrating the hypermethylated and hypomethylated DMRs between ESCA tissues and adjacent tissues by unsupervised clustering. **(D).** Heatmap illustrating the differentiated CpG sites in the DMRs between ESCA tissues and adjacent tissues. **(E).** Principal component analysis of the DMRs between ESCA tissues and adjacent tissues. Abbreviations: DMR, differentially methylated region; ESCA, esophageal cancer.

**
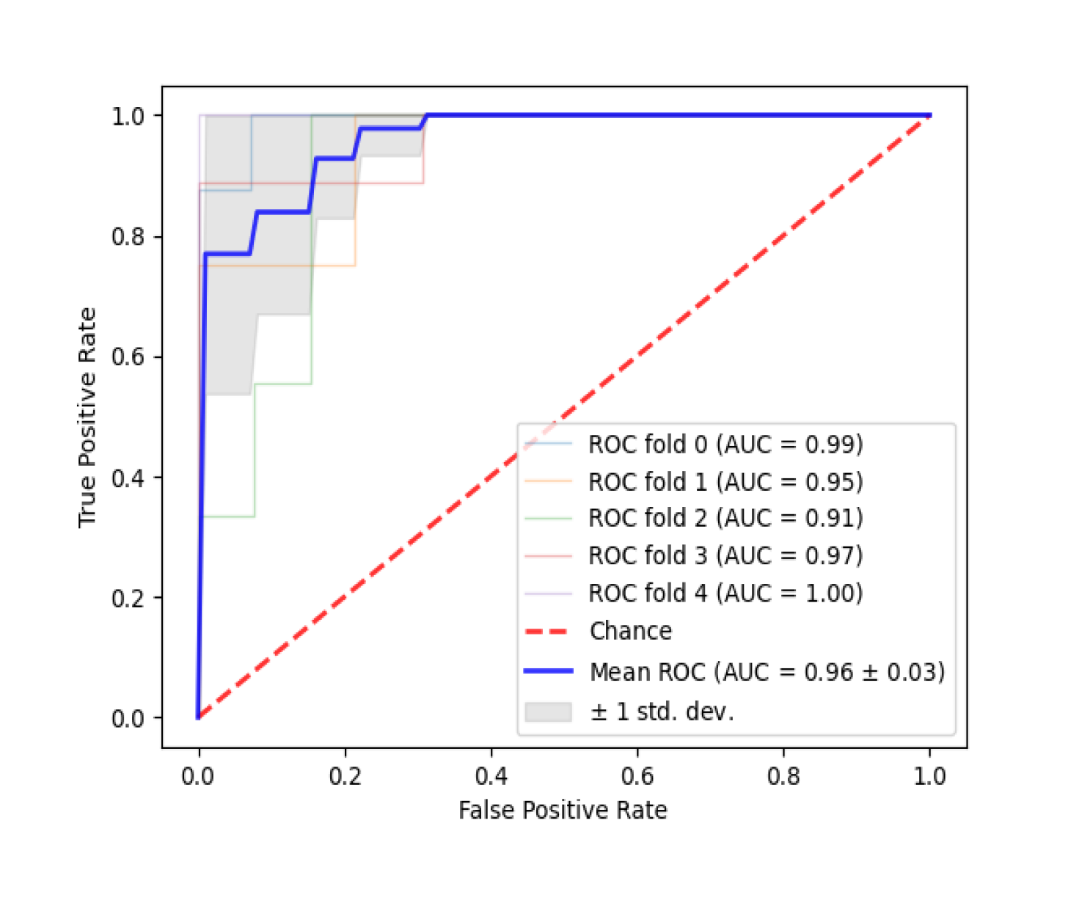
**

**Figure S2. Early detection model development.** Receiver operating characteristic curves by 5-fold cross validation in the training set.


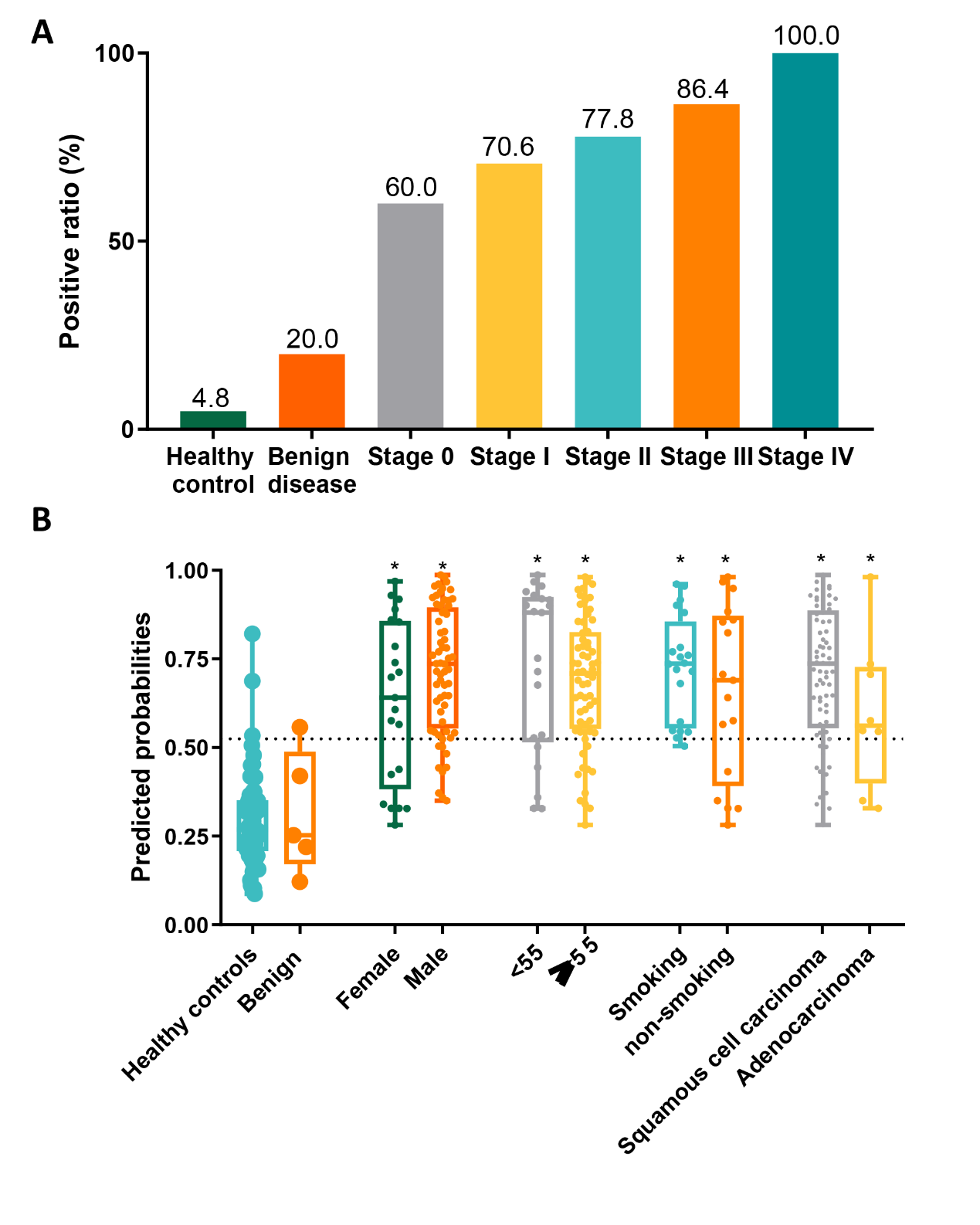


**Figure S3. Performance of the early detection classifier in the total matched population.**

**(A).** Positive ratios in healthy controls, benign esophageal diseases and ESCA with different clinical stages in the total matched population. **(B)**. Predicted probabilities of healthy controls, benign esophageal diseases and ESCA stratified by different clinical covariates in total matched population. * P<0.05 (compared with healthy controls). Abbreviations: ESCA, esophageal cancer.

**
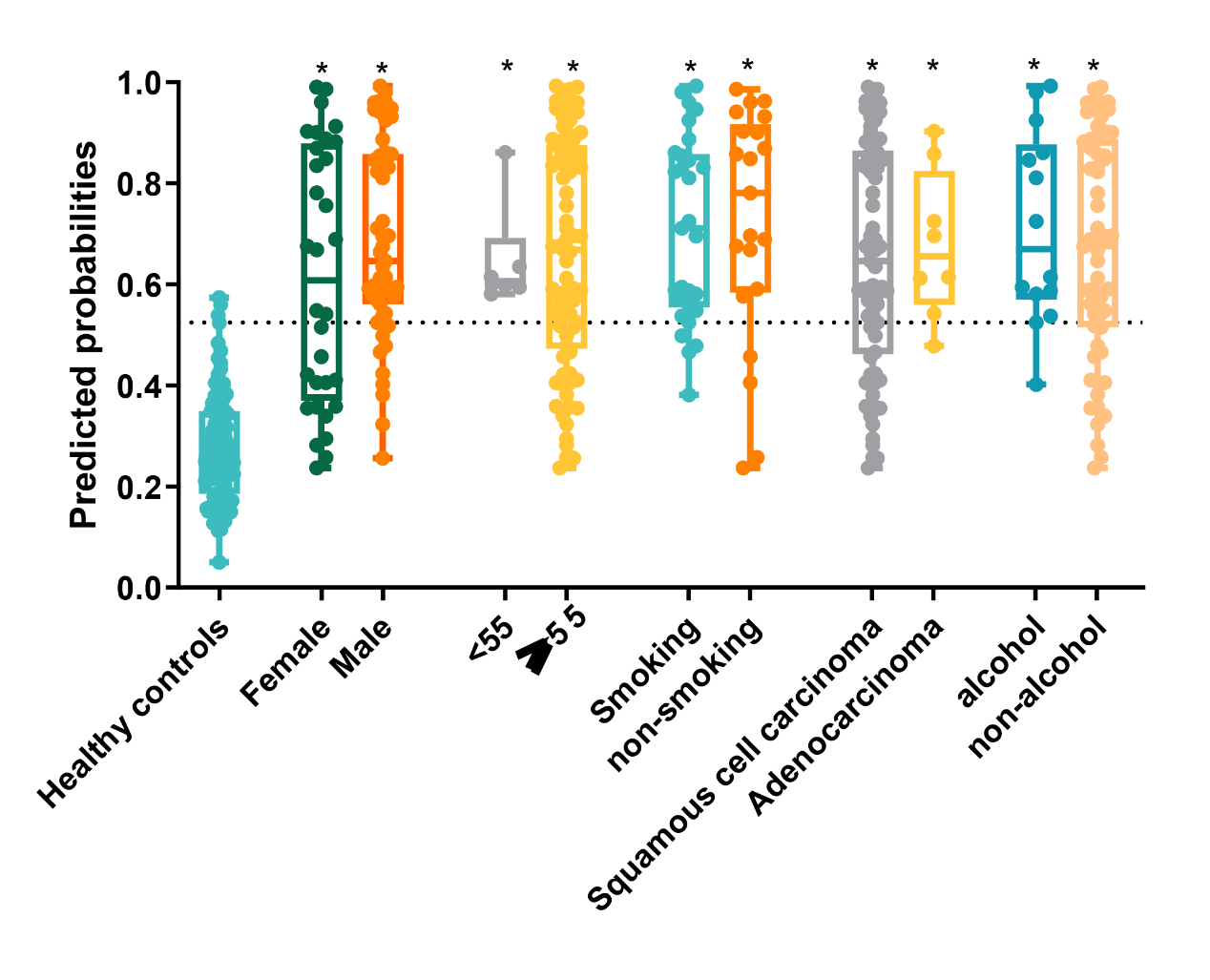
**

**Figure S4. Performance of the early detection model in the validation cohort.** Predicted probabilities of healthy controls, benign esophageal diseases and ESCA stratified by different clinical covariates in the validation cohort. * P<0.05 (compared with healthy controls). Abbreviations: ESCA, esophageal cancer.
